# Design and commissioning of the first superconducting undulator for the BioSAXS beamline at the Australian Synchrotron

**DOI:** 10.1107/S1600577525003418

**Published:** 2025-06-02

**Authors:** Yaw-Ren Eugene Tan, Sina Porsa, David Zhu, Christina Kamma-Lorger, Andrew J. Clulow, Sara Casalbuoni, Andreas Grau, Nicole Glamann, Achim Hobl, Martin Krichler

**Affiliations:** a Australian Synchrotron, ANSTO, Clayton, Australia; b European XFEL Schenefeld Germany; c Karlsruhe Institute of Technology (KIT) Karlsruhe Germany; d Bilfinger, Germany; Advanced Photon Source, USA

**Keywords:** insertion device, synchrotron radiation, superconducting, undulator, cryogen-free

## Abstract

A 1.6 m-long 16 mm-period superconducting undulator (SCU16) has been installed and commissioned at the Australian Synchrotron. The SCU16, developed by Bilfinger, is based on the SCU20 currently operating at Karlsruhe Institute of Technology (KIT). The SCU16 is conduction cooled with a maximum on-axis field of 1.084 T and a fixed effective vacuum gap of 5.5 mm. The design, commissioning experience and performance of one of the longest superconducting undulators at a light source are presented.

## Abstract 2

The performance and experience with one of the first commercially constructed conduction cooled superconducting undulators (SCU16) for the BioSAXS beamline at the Australian Synchrotron are described.

## 1. Introduction

The Australian Synchrotron (LeBlanc *et al.*, 2004 ▸) has been in operation for users since 2005 with nine beamlines to serve Australian and international users. In 2016 the BRIGHT beamline project launched to build eight new beamlines, one of which is the Biological Small Angle X-ray Scattering (BioSAXS) beamline with an undulator source optimized for 12.4 keV located in one of the shorter straights limiting the total insertion device length to under 2.5 m while delivering the same or better flux as the existing SAXS/WAXS beamline with a 3 m undulator.

For a given technology, the minimum allowable vertical gap in the storage ring strongly influences how short a period the undulator could be to maximize the flux at 12.4 keV. A study of the impact on storage ring electron beam lifetime as a function of vertical apertures using existing in-vacuum undulators (IVUs) as well as a pair of vertical scrapers showed that a vacuum gap of 6 mm (at a distance of ±2.0 m either side of the middle of the straight) would impact the lifetime by between 4% and 10%.

**Figure 1:**
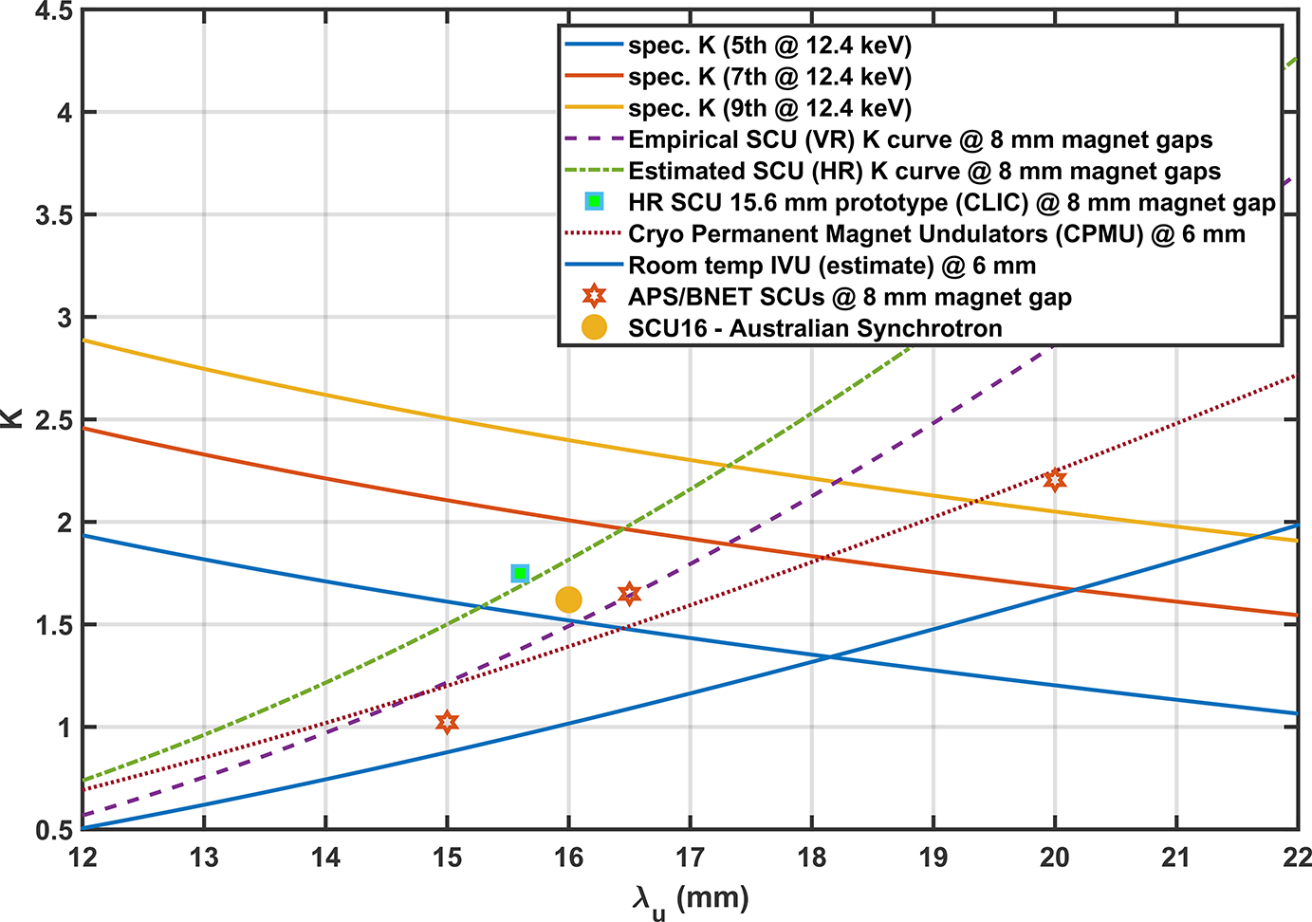
Plot of the relationship between the deflection parameter, *K*, and the undulator period, λ_u_. The three negative sloped curves indicate combinations that result in undulator harmonics at 12.4 keV. The fifth harmonic at 12.4 keV was used to determine the choice of period. The four positive sloped curves represent the different technologies assuming a 6 mm magnet gap (CPMU and room-temperature IVU) and a corresponding 8 mm magnet gap for SCUs where a beam chamber is required. The two curves for the SCU represent the estimated fields for vertical and horizontal racetrack (VR/HR) configurations. The data point for the 15.6 mm period was from Bragin *et al.* (2018 ▸); APS and KIT/Bilfinger data are from Kasa *et al.* (2022 ▸) and Gluskin & Mezentsev (2020 ▸).

Based on known room temperature IVU performances and empirical equations by Bahrdt & Gluskin (2018 ▸) the optimal period for the various technologies was determined to obtain a fixed energy of 12.4 keV. Assuming the use of the fifth harmonic and a total length of 2.5 m a comparison of various undulator technologies was evaluated in Fig. 1 ▸ and Table 1 ▸, in particular cryogenic devices^1^. Under consideration were cryogenic permanent multipole undulators (CPMUs) and superconducting undulators (SCUs). CPMUs are IVUs designed to operate with the permanent magnets cooled to temperatures under 150 K proposed as far back as 2004 (Hara *et al.*, 2004 ▸) and have been in operation at many light sources (Tanaka *et al.*, 2006 ▸; Benabderrahmane *et al.*, 2013 ▸; Bahrdt *et al.*, 2019 ▸; Lu *et al.*, 2017 ▸). SCUs are superconducting electromagnetic undulators that have been used at light sources as far back as 1980 and are in operation at Karlsruhe Institute of Technology (KIT) and Advanced Photon Source (APS) (Gluskin & Mezentsev, 2020 ▸; Kezerashvili *et al.*, 1992 ▸; Ivanyushenkov *et al.*, 2018 ▸; Casalbuoni *et al.*, 2018 ▸; Kasa *et al.*, 2022 ▸) and come in two styles – horizontal and vertical racetracks. The horizontal racetrack (HR) approach follows typical electromagnet wigglers in the past where the wires are wound around an iron pole (perpendicular to the undulator field) which is then assembled to form an undulator. The vertical racetrack (VR) approach is where a single long wire is wound around a long grooved iron former (parallel to the undulator field). There are pros and cons to both styles (Gluskin & Mezentsev, 2020 ▸).

Under ideal conditions and taking into consideration total magnet length differences due to transition requirements, cryogenic devices will provide an expected improvement in the flux density to the beamline by up to 43% with a shorter period device made compared with a room temperature IVU (for the same total length).

The choice of technology came down to flux, maturity of the technology and commercial availability. On paper, CPMUs would be ideal; however, at the time (2019) no proven commercial CPMUs were being offered and research partnerships were untenable, so CPMUs could not be considered. The next consideration was the ability to reach the flux required given the impact of field errors which are quantified by calculating the root-mean-square (RMS) phase error of the magnetic field (Tanaka, 2018 ▸; Walker, 2013 ▸). The phase error is a measure of the difference in arrival time of the electrons relative to the emitted synchrotron radiation at the magnet poles. IVUs and CPMUs have historically demonstrated the ability to achieve small RMS phase errors (<5°) even at periods of less than 20 mm and lengths up to 3 m by shimming (mechanical adjustments of the individual pole position) or sorting individual poles (Chavanne & Elleaume, 1995 ▸). New techniques like force compensation with springs (Huang *et al.*, 2021 ▸) continue to make this possible with shorter periods and smaller gaps. Pole adjustments for SCUs is theoretically possible for horizontal racetrack SCUs (Bragin *et al.*, 2018 ▸); however, it has yet to be demonstrated in practice. By far the most common are vertical racetrack SCUs that rely on tight mechanical tolerances to maintain low field errors as demonstrated at the APS (Kasa *et al.*, 2016 ▸). This is still very challenging, especially to achieve phase errors under 5° for a 16 mm-period undulator. Therefore *SPECTRA* (Tanaka, 2021 ▸) was used to model the expected impact on the photon spectrum in the presence of field errors and concluded that in the worst case scenario RMS phase errors up to 10° could be tolerable with a potential 10% reduction of the flux at the fifth harmonic. With the successful development and demonstration of the conduction cooled SCU20 at KIT (Casalbuoni *et al.*, 2018 ▸; Grau *et al.*, 2018 ▸) in collaboration with Bilfinger GmbH^2^, an SCU was selected as the best source for the beamline in 2019. Parameters of the Australian Synchrotron’s SCU16 are listed in Table 2 ▸.

## 2. SCU16 design

**Figure 2:**
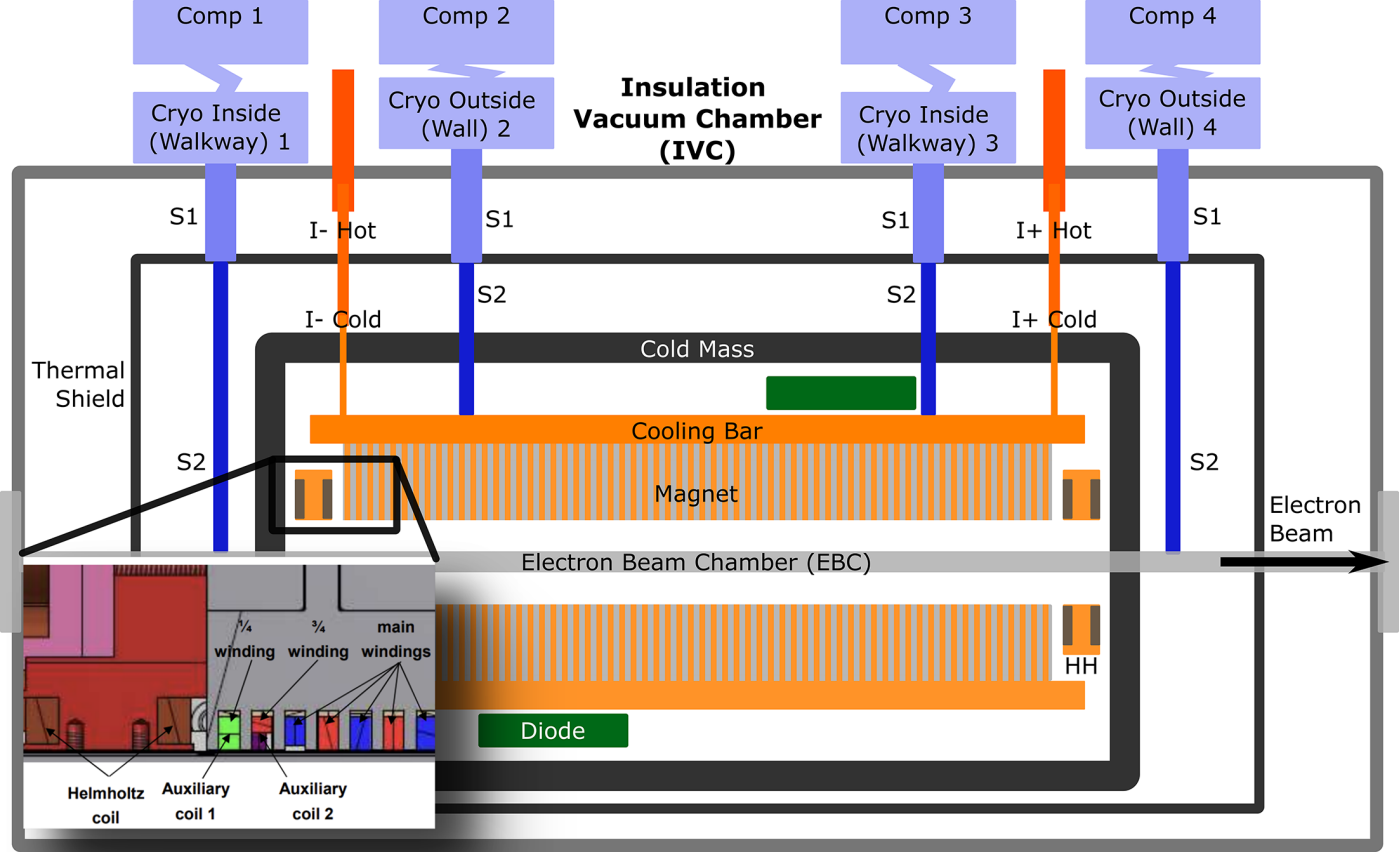
Schematic of the SCU16 with the inset showing the additional windings (AUX1 and AUX2) used for field correction as well as the horizontal Helmholtz coils (HH) for integral corrections.

The SCU16 was designed and built by Bilfinger based on the SCU20 (Casalbuoni *et al.*, 2018 ▸) with a vertical racetrack and Furukawa NbTi insulated superconducting wire [Nb-(47 ± 1) wt%Ti] continuously wound around a 109 mm-wide iron core. The conductor operates at 83.9% of the 4.2 K short sample limit with a current sharing temperature of 5.25 K, above which the conductor is no longer superconducting. The quenching process is the transition from superconducting to normal conducting state and is caused when sufficient energy is deposited locally in the conductor. The lower the operating temperature below 5.25 K, the more robust the SCU16 will be against quenching. In practice, the magnet reaches temperatures of 3.5 K giving a margin of 1.75 K at full field and 200 mA in the storage ring. Additional windings around the end poles (AUX1 and AUX2) along with a pair of superconducting upstream and downstream horizontal racetrack Helmholtz coils (HH) are used for integral corrections. The entire magnet assembly (cold mass) is contained within a larger insulation vacuum chamber (IVC) and is shown in Fig. 2 ▸.

The cryogen-free and conduction cooled design is a key feature, using four Sumitomo cryocoolers (two RDE-412D4 and two SRDE-418D4). The static heat load at 4 K was estimated to be 1.03 W and an additional inductive heating from hysteresis/AC losses of 1.14 W during the ramp up to full field. At 20 K and 50 K the calculated heat load from conduction, radiation and resistive heating was 130 W plus electron beam induced effects up to 6 W and synchrotron radiation heating up to 3 W. With this design the cool-down time was four days as seen later in Fig. 5. A pair of 200 W heaters have been added to reduce the warm up period to 2.5 days. All temperatures are monitored with a combination of 36 Si-Diode and Cernox sensors connected to three Lakeshore 224 units.

### 2.1. Electron beam chamber

**Figure 3:**
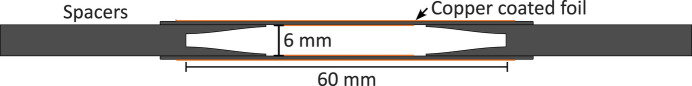
The internal dimensions of the chamber are 60 mm (H) by 6 mm (V). The 316LN stainless steel foils are 0.6 mm thick with 30 µm of galvanized copper on the inner and outer surfaces to increase thermal conductivity and help reduce the resistive wall impedance. The final vertical inner aperture should be 5.94 mm.

The electron beam chamber (EBC) made from 316LN stainless steel was re-designed to increase the robustness under one atmosphere of differential pressure without significant and lasting deformations, which is sometimes required during annual maintenance of the cryocoolers. A cross section of the EBC is shown in Fig. 3 ▸. The internal dimensions of the chamber are designed to be 60 mm (H) by 6 mm (V) with the inner upper and lower surfaces coated with an additional 30 µm of galvanized copper to improve electrical and thermal conductivity. The large horizontal dimensions of the electron beam chamber were required to ensure that the upstream dipole synchrotron radiation was not intercepted by the vacuum chamber. The total external height is 7.2 mm, giving a 0.4 mm gap between the magnet and chamber surface. When cooled, the total length of the EBC contracts by 7.5 mm.

### 2.2. Power supply and quench protection system

Three Delta Elektronika SM 15–400 (15 V/400 A) power supplies arranged in parallel (one master, two slaves) are used to power the SCU16, each with a rated 8 h stability of 100 p.p.m. The primary passive quench protection system is a set of cold diodes that protect the superconductors coupled with Danfysik’s four-channel quench detector (System 8500) used to monitor the voltage drop across two sections (magnet and high temperature superconductor). The power supply is interlocked if a quench condition is detected, *i.e.* a differential voltage exceeding 100 mV for more than 10 ms, or if magnet temperatures are above 4.25 K. The static power consumption of the SCU16 (including cryocoolers) is approximately 30 kW.

### 2.3. Field measurements

Magnetic measurements of the SCU16 were performed with just the cold mass (magnet and supports) at KIT’s CASPER II facility (Grau *et al.*, 2019 ▸; Grau *et al.*, 2016 ▸) with a stretched wire system for integral measurements and hall sensor measurements with a sledge for longitudinal field profiles. Low temperature Hall sensors mounted on a brass sledge, guided precisely in the middle of the gap height, were used for local field measurements. The sensors were calibrated at 4 K, and from the fitting procedure of the field to voltage calibration the standard deviation of the data leads to a measurement precision of 100 µT. By shifting the Hall sensor 10 mm horizontally and repeating the longitudinal field profile measurement, the roll-off can be calculated. To ensure a precise position determination of the sledge below 1 µm, a laser interferometer was attached to CASPER II and targeted through a window to a retro-reflector mounted on the sledge that measures the position while moving the sledge step-wise along the undulator coils. To power the coils a polarity changeable Bruker 1500 A power supply with a current stability of 1 × 10^−5^ was used during training and field characterization. Training quenches were detected by a quench detector designed and manufactured at the Institute for Data Processing and Electronics (IPE) at KIT and the quench diagnostics were performed via a 64-channel, 200 kHz data acquisition system from National Instruments run by a *LabView* program.

**Figure 4:**
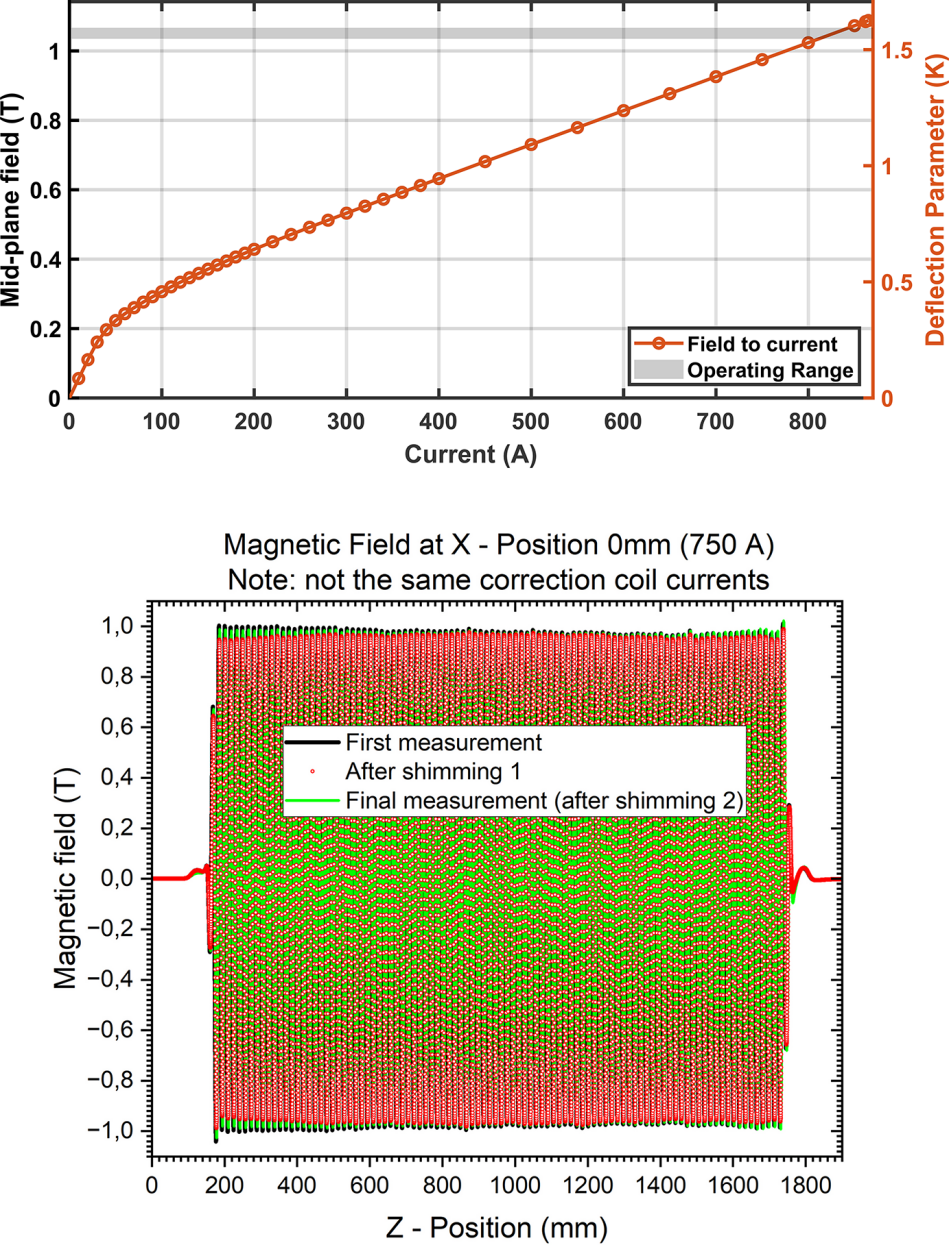
(Top) Measured midplane field and deflection parameter, *K*, at a pole as a function of current. (Bottom) Comparison of fields before and after adjusting the gap using shims as measured on the midplane.

Technical issues, COVID delays and other time constraints meant that only two iterations of adjustments (by adding shims) of the spacers that define the gap between the two magnetic arrays could be performed to symmetrize the transverse roll-off and minimize the field amplitude variation along the length of the SCU. With the current corrections the RMS phase error is estimated at an equivalent of 10°. With additional mechanical shimming it would have been possible to reduce the phase error to less than 6°, as discussed in later sections. The results of these field measurements are shown in Fig. 4 ▸. The RMS phase and integral errors are shown in Table 3 ▸.

## 3. Commissioning

**Figure 5:**
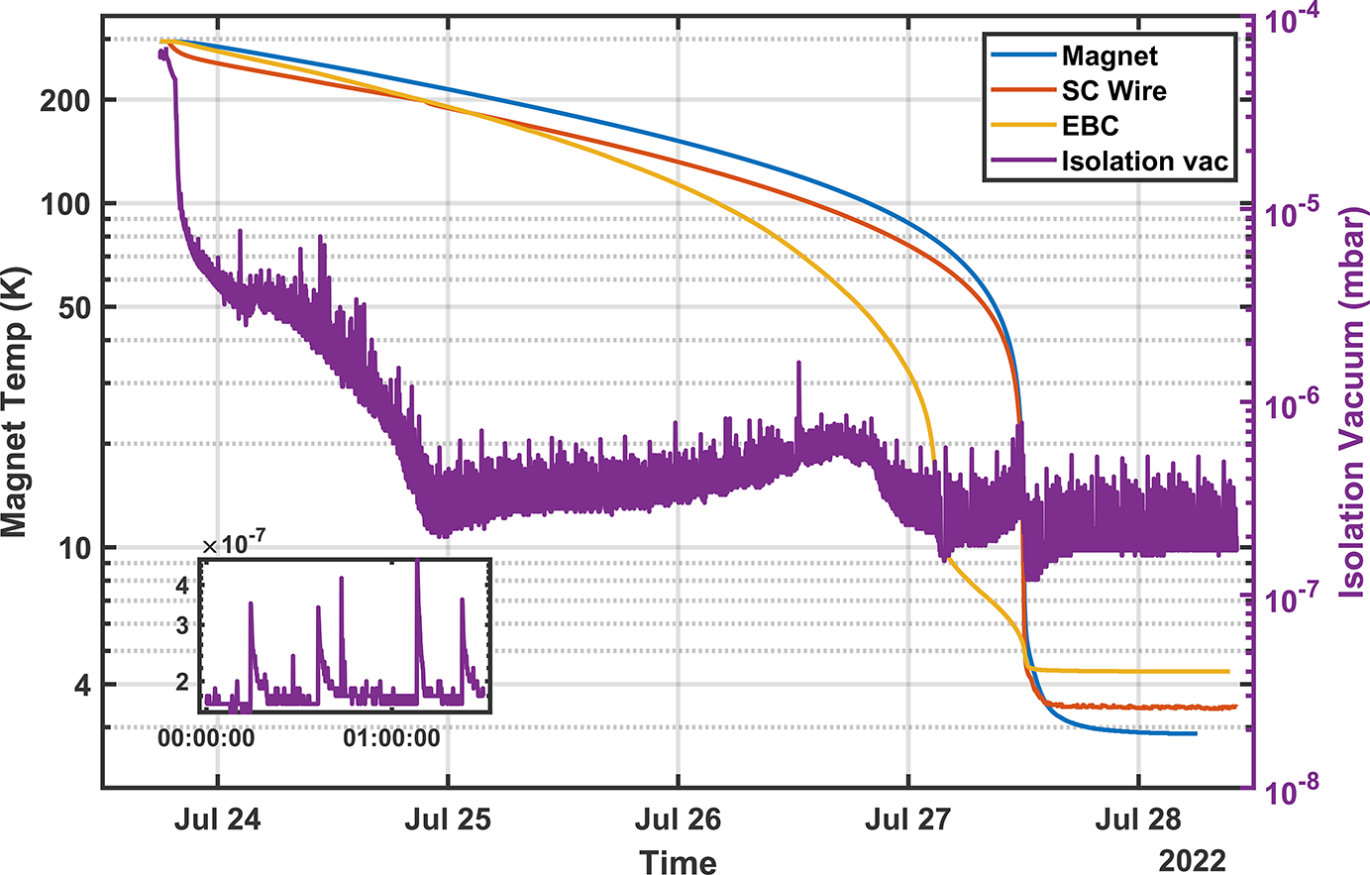
Cool down takes just under four days. Small vacuum spikes (inset) are likely the result of out-gassing from various trapped volumes within the IVC.

Cooldown took just under four days and resulted in a final IVC pressure of less than 10^−6^ mbar (see Fig. 5 ▸). Below 10^−5^ mbar, periodic pressure spikes emerged, believed to be the result of trapped volumes in elements like the layered Mylar thermal shield; however, after a few months these spikes have largely disappeared.

**Figure 6:**
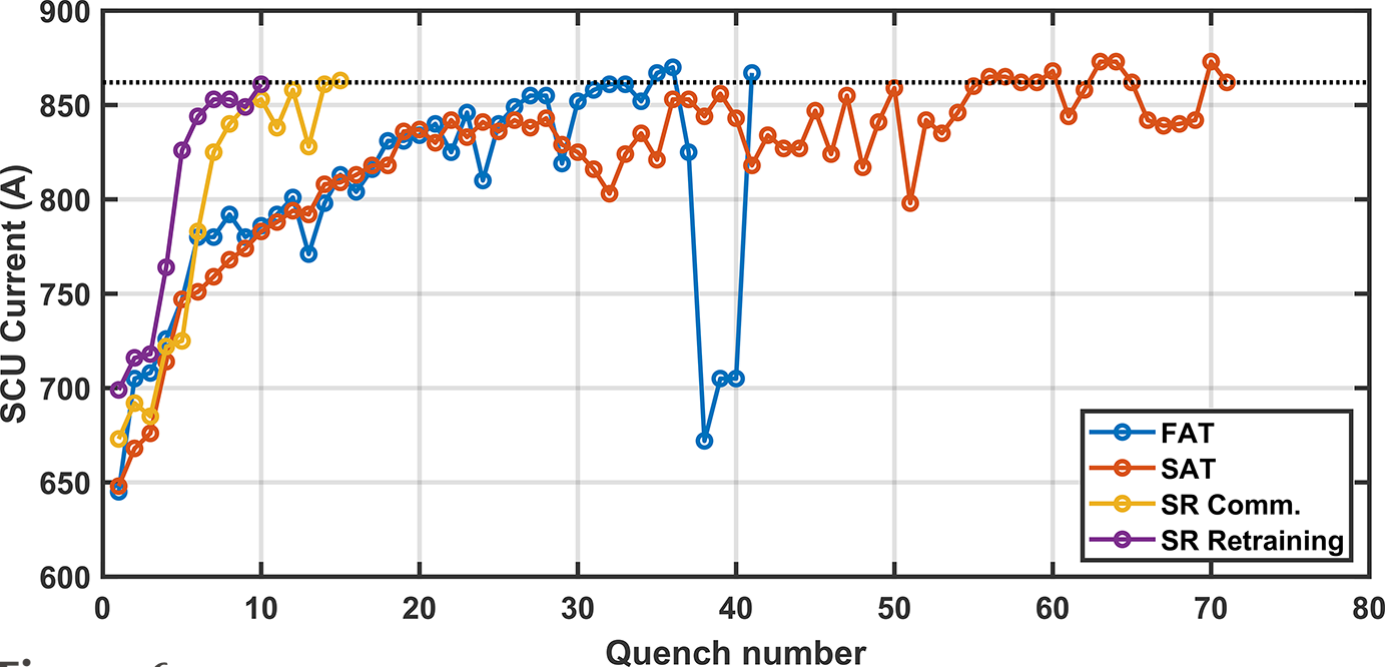
Quench history to the nominal operating current of 862 A (1.084 T) during factory acceptance (FAT), site acceptance (SAT), commissioning in the storage ring and re-training after warm up.

**Figure 7:**
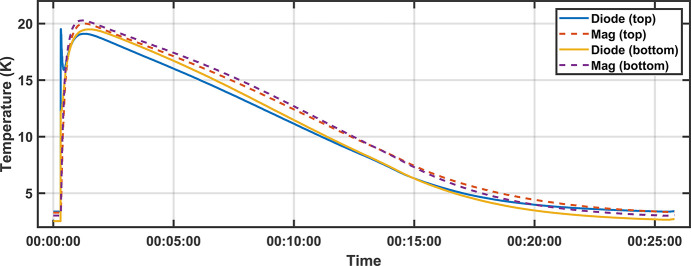
After a quench, magnet temperatures reach approximately 20 K followed by recovery taking 25 min.

Fig. 6 ▸ shows the number of quenches required for training to reach the nominal field of 1.084 T (862 A). The increase in the number of quenches required during Site Acceptance Testing (SAT) is likely a combination of transport (recorded minimum/maximum temperature of 10°C/30°C and a maximum shock value of 16 g) and opening of the IVC on site for adjustment of the vacuum chamber. The longer training schedule took four additional days to complete. Subsequent trainings have been very quick, taking less than 10 h to complete, indicating no permanent impact to the system. With a maximum ramp rate of 4.5 A s^−1^, full field is reached in 3 min. After each quench the magnet and diode temperatures reached 20 K before cooling down to operational temperatures after 25 min, as shown in Fig. 7 ▸. In one incident, a mis-configuration of the PSU resulted in the current output that was a factor of 1.33 greater than reported, and resulted in the SCU16 trained to a field of 1.12 T (*K* = 1.673). The experience reinforced the need for an independent direct current current transformer (DCCT) to monitor current.

**Figure 8:**
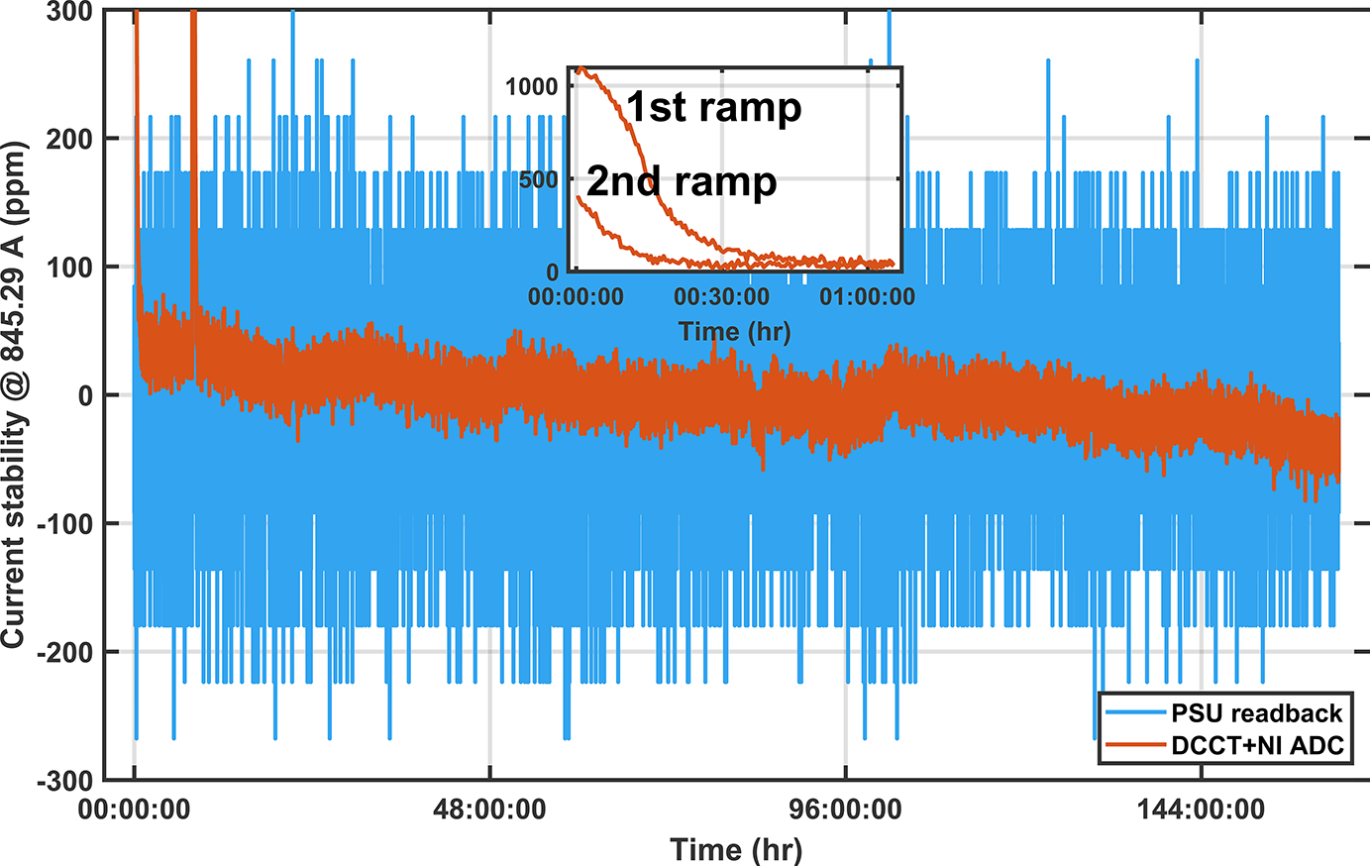
Current stability measured with an independent DCCT over 160 h time period at 1.07 T (845.29 A). Two ramps spaced 5 h apart show the power supply returning to the same current with slightly shorter warm up time. PSU warm up of 30 to 60 min (inset). After warm up the r.m.s. and peak-to-peak current stability were 19 p.p.m. and 170 p.p.m., respectively. The monotonic drift of 80 p.p.m. is likely due to the temperature dependence of the power supply rated at 60 p.p.m. °C^−1^ and local rack temperatures are stable to ±1°C.

An external DCCT (Ultrastab 866R) differentially sampled with a 16-bit ADC (NI-6221)^3^ measured the SCU current independently and the results plotted in Fig. 8 ▸ show an RMS and peak-to-peak field stability of 19 p.p.m. and 170 p.p.m., respectively, over a 160 h time window during normal operation. The results also show the warm up period (up to 45 min) that could be as large as 1000 p.p.m. before reaching the final stable operating current (inset of Fig. 8 ▸) as well as a slower monotonic drift of the output current of 200 p.p.m. over two weeks. Both exceed the original stability requirement and would require a feedback system; however, after a year of operation none have been needed so far.

During site acceptance prior to installation, a stretched wire system developed by KIT was used to verify that the field integrity had not been damaged by shipping. The first and second integrals were measured as a function of horizontal offsets (±1 mm, ±3 mm, ±5 mm) and the results compared with those in Table 3 showed a maximum difference of 4 mT, 49 µT m and 741 µT m for the integrated quadrupole, and first and second integrals, respectively (the earth field contribution of 106 µT m and 169 µT m removed). The results were within our expectations given the uncontrolled environment it was measured in and that a fault in any of the coils would have resulted a large increase (>1000) in both first and second integrals while assembly movements would also affect the integrated quadrupole component.

**Figure 9:**
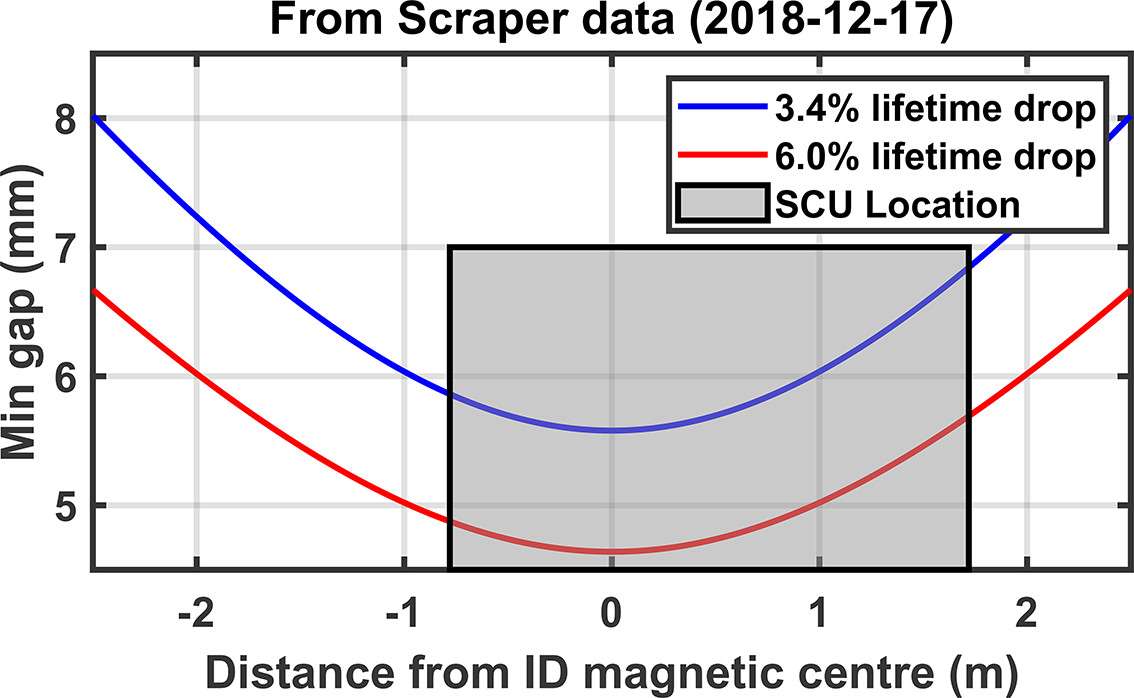
Estimated impact on lifetime due to different vertical apertures as measured with scrapers and confirmed with existing IVUs. Measurements were made in 2018 and were one of the key factors in determining the minimum gap requirement.

The same system was used to verify the vertical aperture by touching the wire to the vacuum chamber’s top and bottom surfaces. After making some adjustments to the end flanges the parallel gap was measured to be no larger than 5.6 mm (taking into account measured wire tension, diameter, density and resulting sag). Though narrower than our requirement of 6.0 mm it has had minimal impact on the lifetime. Table 4 ▸ lists a comparison of the measured lifetime just after a regular maintenance shutdown, with the storage ring in the same configuration showing a reduction of <2% after the SCU16 installation and no noticeable impact on injection efficiencies, in line with expectations of 3% based on scraper measurements shown in Fig. 9 ▸.

**Figure 10:**
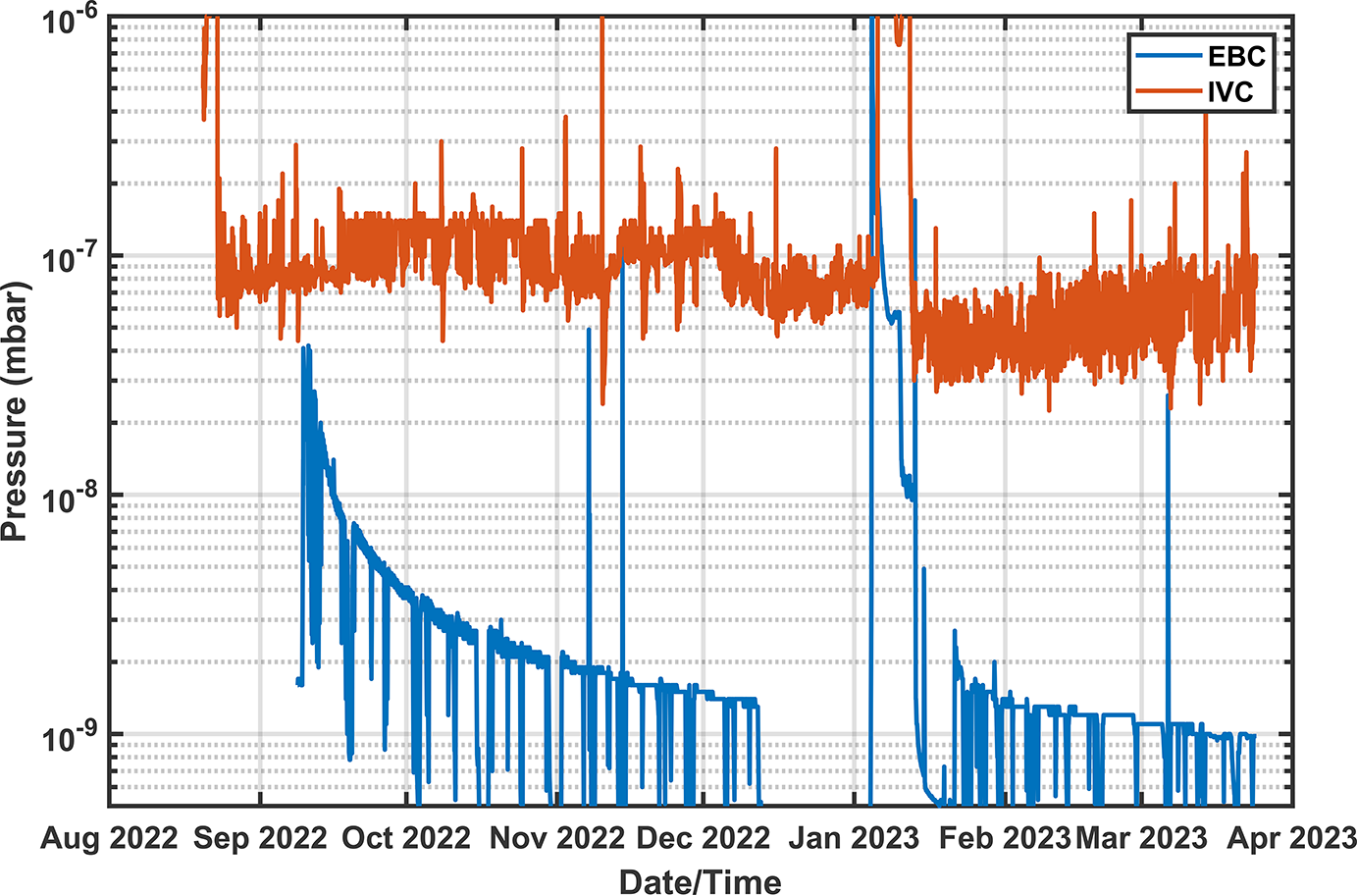
Vacuum conditioning of the EBC and IVC. In January 2023 the SCU was allowed to warm up naturally while being pumped to remove excess gases cryo-pumped during operations. Only an improvement in the EBC vacuum was detected.

**Figure 11:**
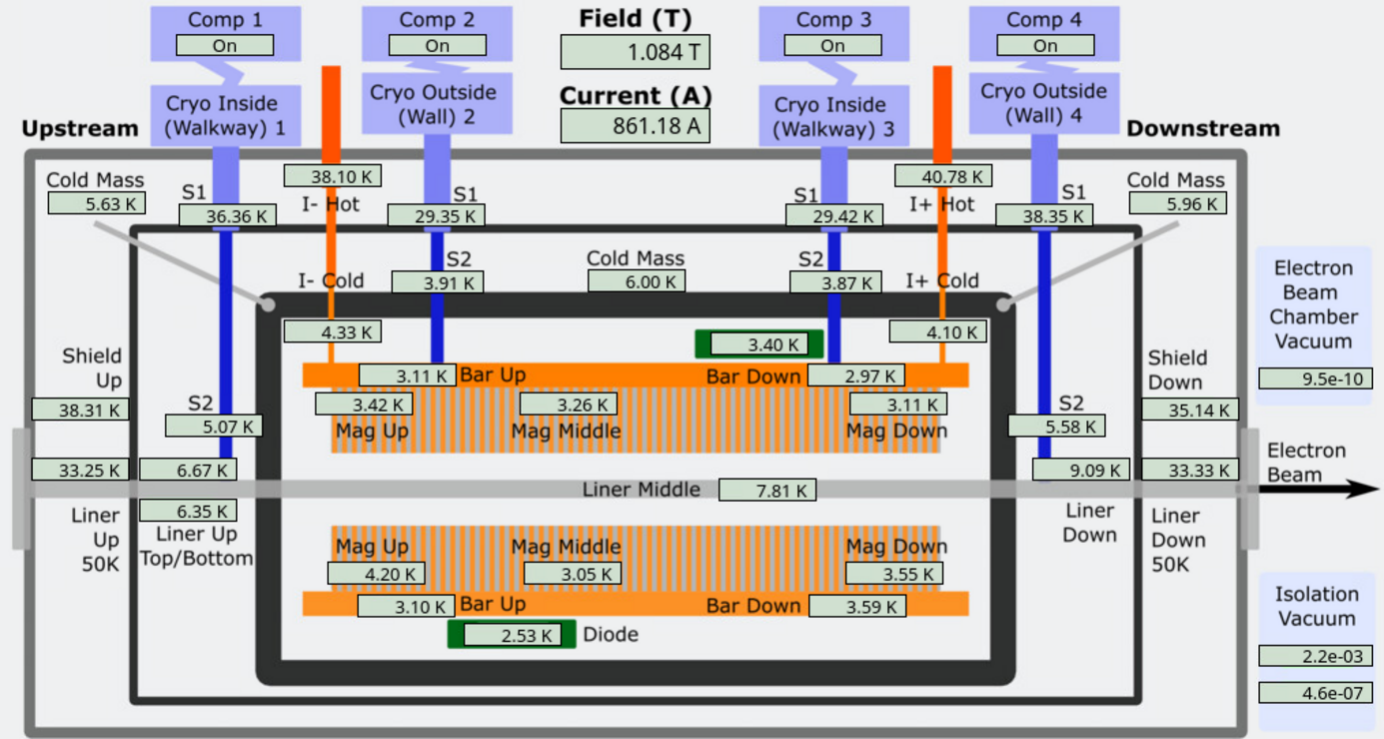
Control system graphical user interface showing the steady state temperatures for the SCU16 at 1.084 T (maximum field) and 200 mA in the storage ring.

Vacuum conditioning after first beam was smooth reaching mid-10^−8^ mbar after a few days, and has since reached below 10^−9^ mbar (Fig. 10 ▸). The equilibrium temperatures of the SCU16 at 1.084 T and 200 mA in the ring are shown in Fig. 11 ▸ with magnets reaching temperatures of <3.5 K. The temperature distribution at the EBC also shows elevated temperatures at the downstream end consistent with additional heat load from the upstream dipole synchrotron radiation. The downstream EBC temperature can fluctuate up to 2 K as week to week conditions change.

**Figure 12:**
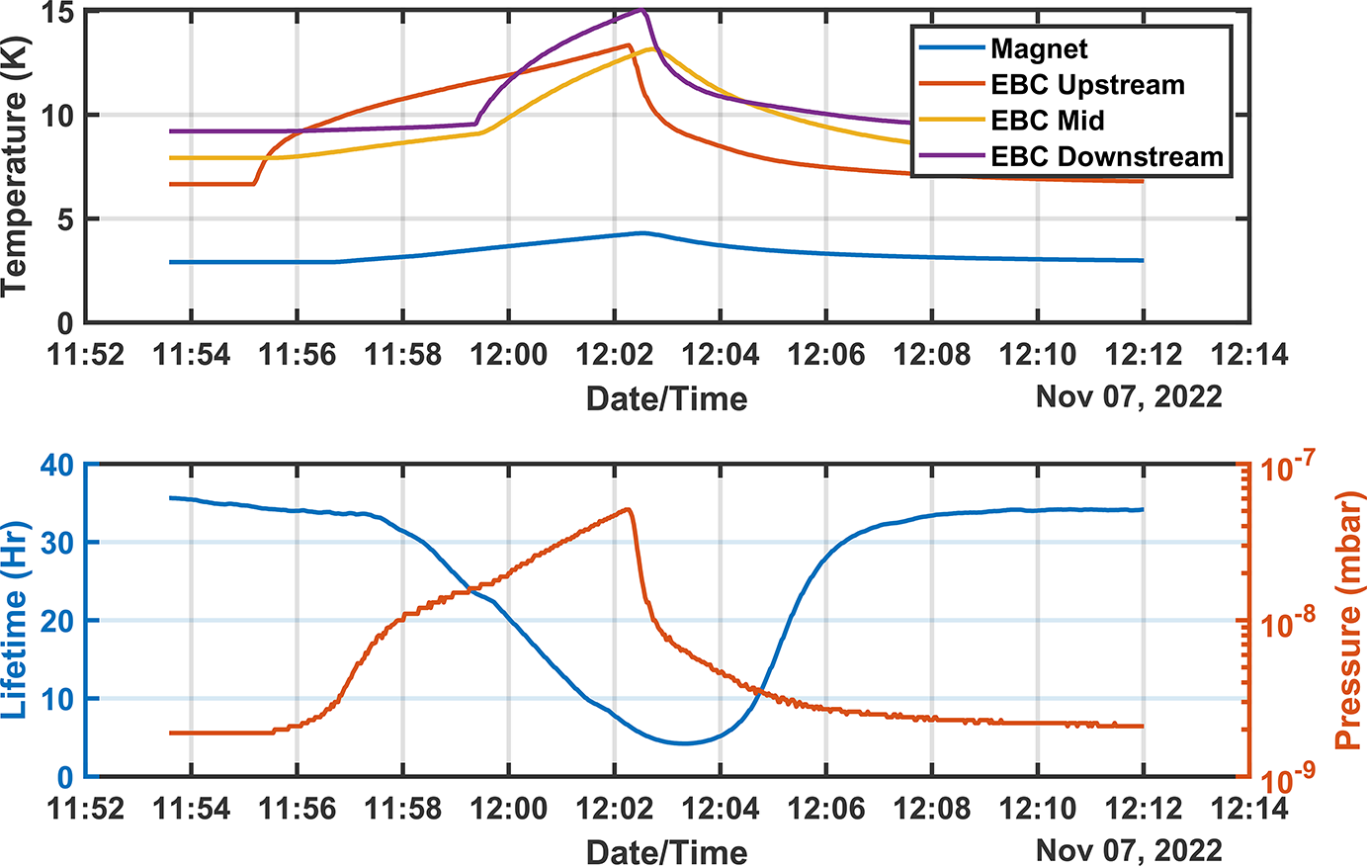
The EBC vacuum chamber pressure increases rapidly by more than one order of magnitude in 5 min if all the compressors are turned off with 200 mA in the storage ring, most likely from the desorption of hydrogen and nitrogen above 8 K. A pressure monitor (cold cathode) is located downstream of the EBC and true pressures internal to the EBC are likely to be one order of magnitude above those measured here. The lifetime drops extremely rapidly to less than 5 h within 5 min.

One significant risk of a cryogen-free system is the rapid temperature rise in the absence of the cryocoolers. This was evident when cooling water was disrupted causing the cold-heads to overheat and shut down for 30 min. In a study to determine the effect of this failure mode with stored beam the compressors were turned off for 8 min and resulted in a rapid increase in pressure in the narrow gap chamber as shown in Fig. 12 ▸. The higher pressure resulted in a sharp decline in the lifetime after 5 min and evidence of vertical beam instabilities was also observed. It was clear from this test that a disruption to the cooling water would result in a beam dump. To ensure continuous operation, the cooling water is monitored and will switch to a secondary cooling system in the event of a disruption to keep the compressors running.

Over the first six months of operation, the SCU16 has demonstrated a quench rate of 5.6% (one quench from 18 beam dumps). By comparison, our 4.2 T superconducting wiggler (SCW) has a quench rate of 71% (25 out of 35 beam dumps). In all cases, quenching of the SCU16 has been attributed to a beam dump. Tests have confirmed that the quenches during a beam dump are the result of scattered particles generated by 3 GeV electrons interacting with the SCW, and to a much lesser extent the SCU16. A more controlled beam dump that directs the electron beam into a scraper in a shielded location is being implemented to eliminate beam dump related quenches. Since commissioning, quench rates have not changed significantly.

### 3.1. Impact on closed orbit and tunes

**Figure 13:**
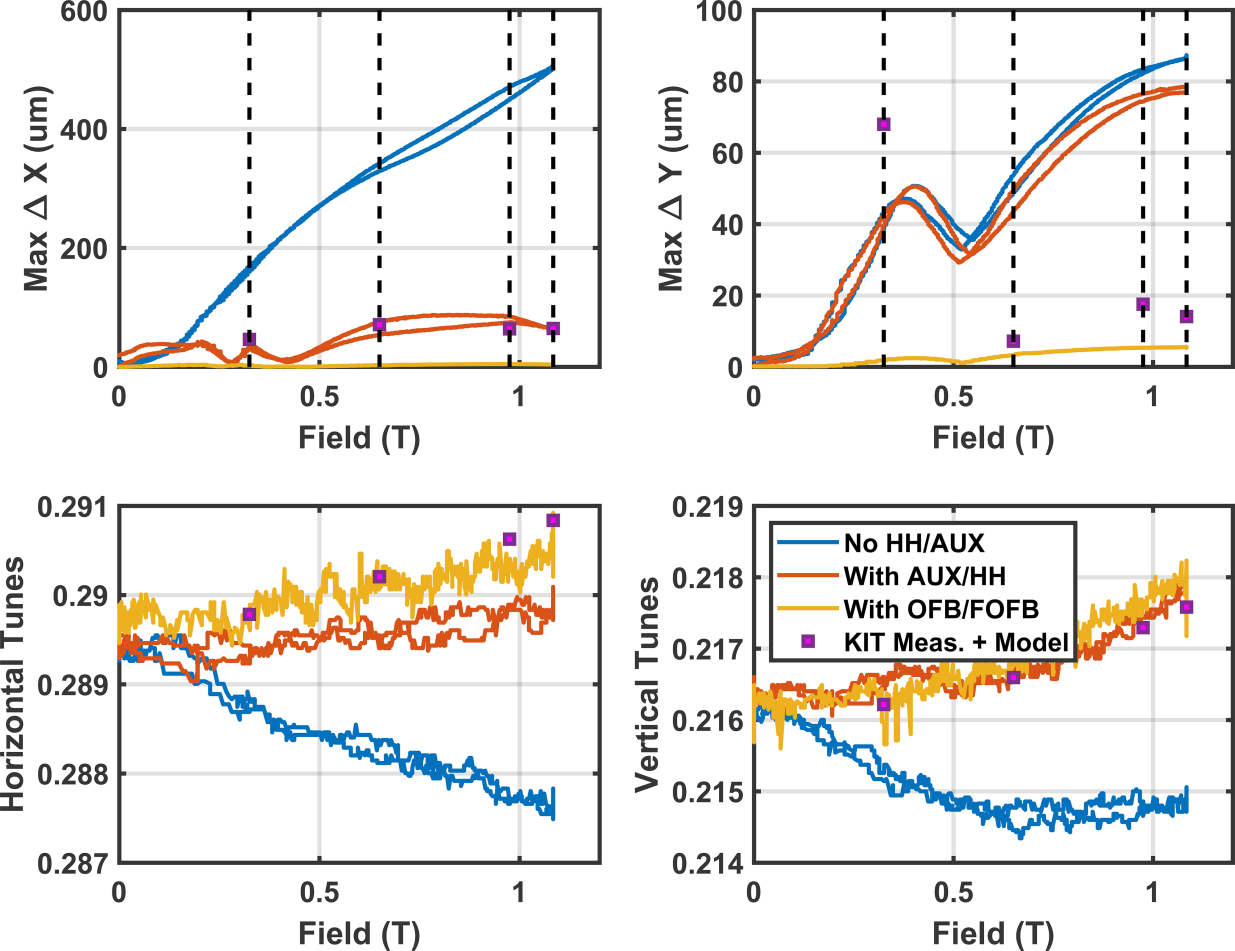
Closed orbit disturbance during ramp up and down from nominal operating current with (red) and without (blue) corrections. When orbit feedback is included the maximum orbit perturbation is under 5 µm. With minimized orbit perturbations the maximum tune shift is Δν_*x*_/Δν_*y*_ = 0.001/0.002. The squares are the expected orbit perturbation and tuneshifts based on the measured magnetic fields, taking into account the change in the contribution of the earths magnetic field as the iron poles saturate and edge focusing in the undulator.

The impact of SCU16 on the maximum closed orbit distortion and tune is shown in Fig. 13 ▸. With the optimized HH coils currents the maximum orbit deviation was kept under 100 µm; with the orbit feedback system (Tan *et al.*, 2017 ▸) it was kept under 5 µm.

From simulations it is possible to derive the closed orbit response to an offset and angular perturbation to the electron beam using Δ*X*, Δ*Y* = 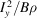, 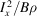 and Δ*X*′, Δ*Y*′ = 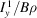, 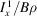, respectively, where *B*ρ = 10.01 is the beam rigidity for a 3 GeV electron beam. A second contribution to take into consideration is the earth magnetic field which increases by a factor of two if unsaturated iron poles with the geometry of the undulator are modeled (8 mm gap). When the SCU16 is at a field of 0.3 T the iron poles are saturated (2 T) and this increases the effective gap to ∼28 mm which decreases the concentration factor to 1.4. With an earth field of 60 µT on site, ramping the SCU16 from zero past 0.3 T results in an additional vertical field of (2.0 − 1.4) × 60 µT over 1.6 m, giving a total *B*_*y*_*I*1 of 57.6 mT m. Taking both the earth field contribution to the vertical integral and residual integrals in Table 3 ▸ the maximum residual orbit perturbation can be calculated, shown as squares in Fig. 13 ▸. The expected contribution to the tune shift from additional focusing from the SCU16 was taken from the measured gradients, *k*, and modeled edge focusing in an ideal undulator. The measured fields from Table 3 ▸ agree very well with measured orbit perturbations and tuneshift, with the exception of Δ*Y* where there is a 60 µm discrepancy. Such an unexpected horizontal field integral was also observed with the HEX SCW (Hidaka *et al.*, 2023 ▸) and may be the result of fields generated by feed cables to the SCU16 or internal wire routing within the device.

**Figure 14:**
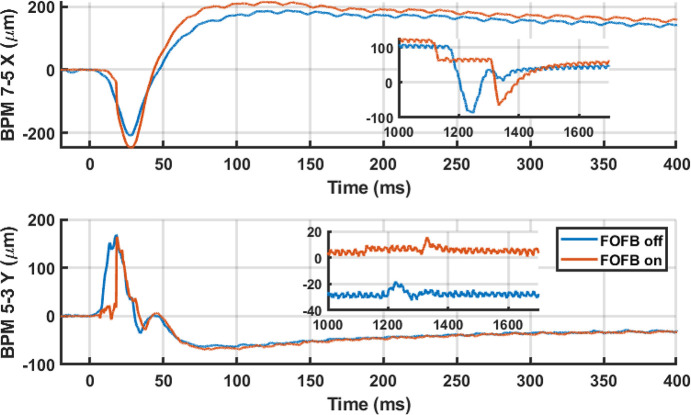
The maximum perturbation to the stored beam was recorded to be 250 µm (H) and 170 µm (V) which is below the orbit interlock limits. The disturbance is, however, too large for the fast orbit feedback system (FOFB) to correct and stops 20 ms after quench. The inset shows the disturbance from the AUX/HH correctors interlocking and turning off.

To collect data on the impact of a quench on the electron beam, a quench was initiated by increasing the field. Fig. 14 ▸ shows a record of the beam position at the BPM with the largest response during a quench. Positions measured at 10 kHz come from Libera Brilliance fast acquistion data (Tan & Hogan, 2018 ▸). The disturbance is too large for the fast orbit feedback system (FOFB), which attempts to correct the fault in the first 20 ms before stopping, but small enough that it does not trigger an orbit interlock to cause a beam dump.

## 4. Photon spectrum

**Figure 15:**
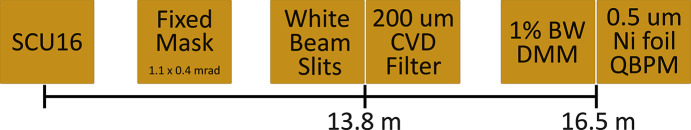
Layout of the BioSAXS beamline related to the current photon spectrum measurement from SCU16.

**Figure 16:**
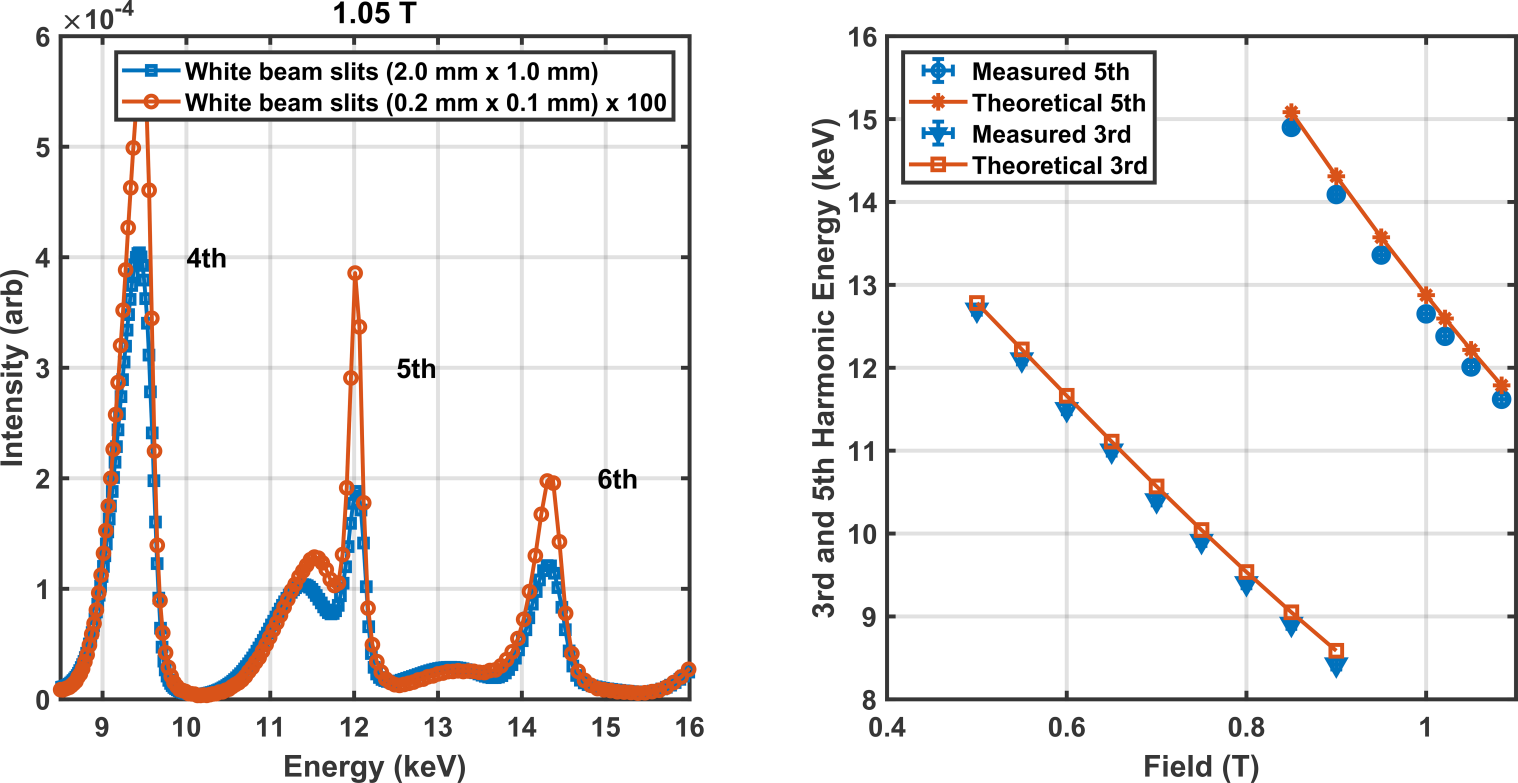
Measured spectrum at a field of 1.05 T which places the measured fifth harmonic peak at 12 keV (left). The intensity takes into account the transmittance of the 200 µm CVD filter and 0.5 µm Ni foil detector. The energies of the third and fifth harmonic peak are consistently below the theoretical and possibly indicate a 1.4% error in the true field. The energy step size of the measurements is 43 eV (0.47% at 9 keV to 0.29% at 15 keV).

The photon spectrum of the SCU16 was measured on the BioSAXS beamline. A schematic of the layout of the optics involved in this measurements is described in Fig. 15 ▸. The photon spectrum was measured using the sum signal from a QBPM (Alkire, 2017 ▸) that detects the fluorescence from a 0.5 µm Ni foil on four photodiodes. Fig. 16 ▸ shows the spectral measurements with white beam slits fully open at 2.0 mm (H) by 1.0 mm (V) and with slits closed to 0.2 mm by 0.1 mm (14.5 µrad by 7.2 µrad). The narrow slits were set to a value smaller than the photon beam divergence of 36 µrad and 14 µrad. The aperture is centered at the location with the greatest sum signal on the QBPM. The calibration of current to field was interpolated from the measured data shown in Fig. 4 ▸.

The spectrum was measured in intervals of 43 eV from 8 keV up to 16 keV for fields from 0.50 T to 1.084 T. The results shown in Fig. 16 ▸ indicate a very large fourth harmonic peak relative to the fifth even after compensating for the effects of the 200 µm CVD diamond and 0.5 µm Ni foil used in the QBPM. In addition, the measured fifth harmonic peak was notably lower by 166 eV when compared with theoretical expectations based on the measured fields. The photon energy was calibrated against the three absorption edges for Cu, Zn and Se with an uncertainty of ±10 eV and is unlikely the cause. The storage ring electron energy was measured with an uncertainty of ±0.01% (Wootton *et al.*, 2013 ▸). The most likely cause is a 1.4% error in the field to current calibration in Fig. 4 ▸. Another possibility is a significant vertical offset. This was also modeled and could explain some of features seen in the measured data.

**Figure 17:**
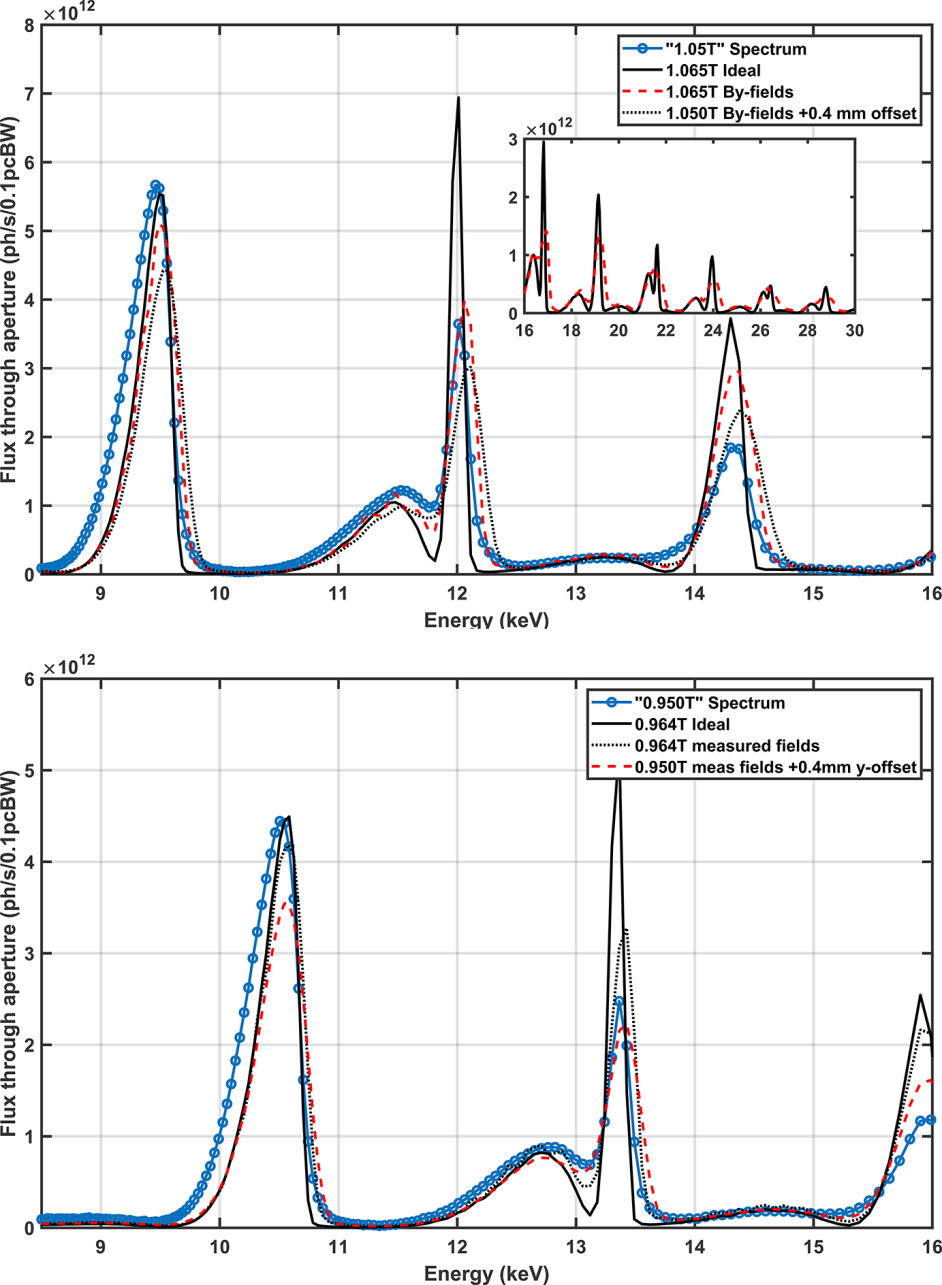
Beamline measurements of the SCU16 spectrum after a DMM with a 1% FWHM bandpass at 1.05 T (top) and 0.95 T (bottom) using the summed signal from a QBPM. The measured spectrum is in amps from the QBPM photodiodes and is scaled to compare against simulation expectations. The magnetic field from 1.084 T was scaled to 1.050 T and 1.065 T, then imported into *SPECTRA* to calculated the photon spectrum. The results imply that either the current for ‘1.05 T’ is in reality 1.065 T or the beamline is significantly vertically misaligned. The same calculation was applied for the 0.95 T data set. The comparison was not conclusive in that either scenario could be the case. The inset shows the calculated spectrum from ideal and measured fields at 1.065 T.

The amplitude of the measured photon spectrum at 1.05 T and 0.95 T has been scaled to compare against the theoretical expectations in Fig. 17 ▸. The numerical approximation of the spectrum was calculated using *SPECTRA* (Tanaka, 2021 ▸) with data from measured magnetic fields. The flux through an aperture, representative of the WB slits, was calculated for a field 1.4% higher than what was set in the control system and also for the reported field and a 0.4 mm vertical offset. Both scenarios appear to replicate the features seen in the measured data; however, an error in the current to field calibration is a better fit and more likely.

**Figure 18:**
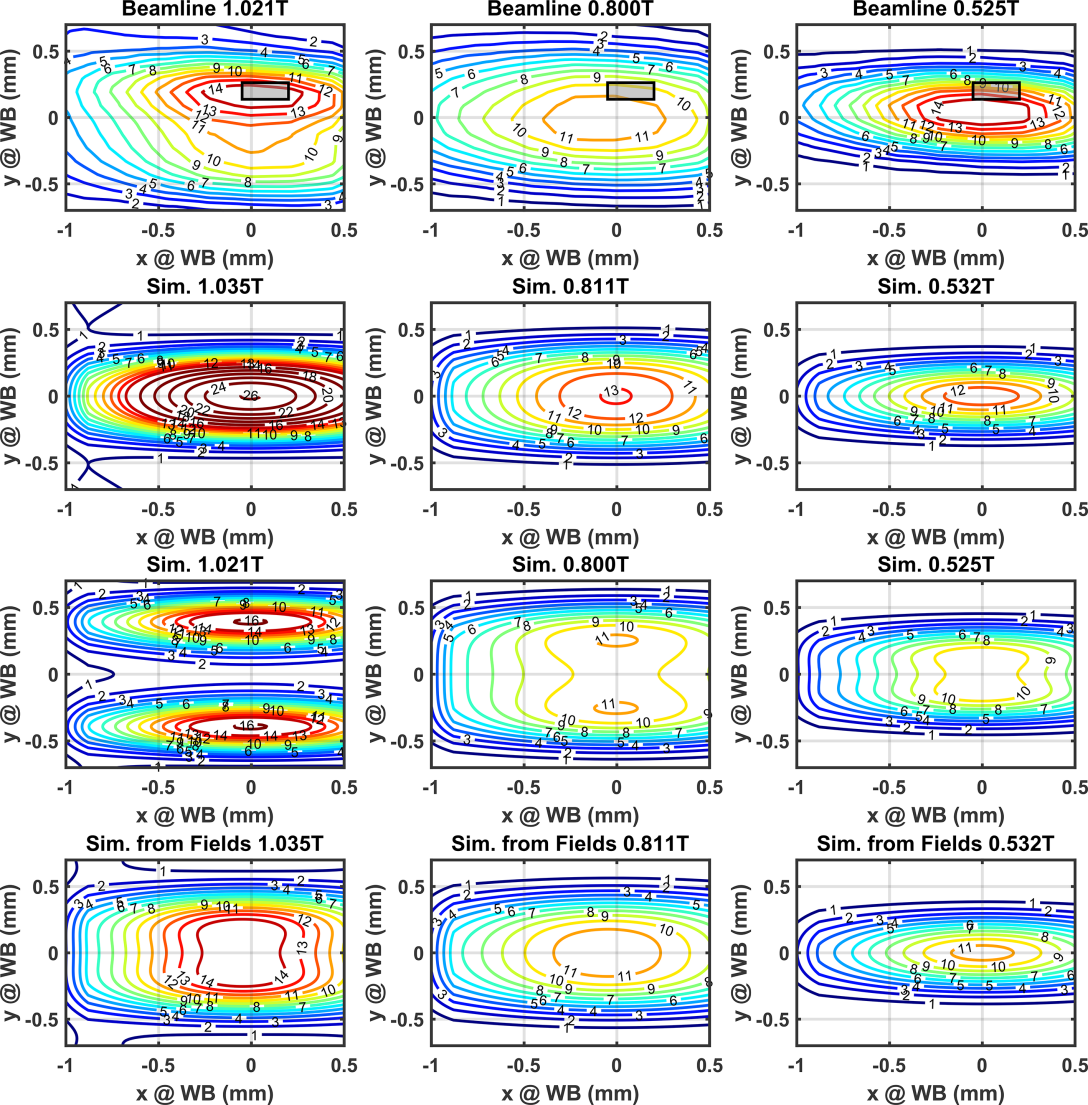
Photon intensity was measured with beamline optics set to a central energy of 12.4 keV and 0.2 mm by 0.1 mm WB aperture (third row). The center of the slits was then scanned over a 2 mm range to map the distribution of the fifth, fourth and third harmonics at 12.4 keV (left to right columns). The gray box shows the nominal center of the WB aperture during previous measurements. The measured data in amps is normalized to the peak of the simulation in the bottom row. Contours in units of 1 × 10^16^ photons s^−1^ mrad^−2^ (0.1% bandwidth)^−1^. Simulation of the ideal spatial flux density through a 0.2 mm by 0.1 mm aperture for the reported field and 1.4% higher field (second and third row). The spatial flux density calculated from scaled magnetic field measurements are shown in the bottom row.

A raster scan of the aperture was made, this time with the double multilayer monochromator (DMM) centered at 12.40 keV ± 0.01 keV to confirm whether the WB slit was vertically misaligned. The raster scan at the top row of Fig. 18 ▸ indicates that the centroid does move up to 200 µm at different field settings. The flux distribution was simulated in *SPECTRA* for both the reported field and a higher field. The simulated photon distribution was integrated to simulate the 1% DMM.

Between the two ideal photon distributions considered, the measured data are consistent with the higher field of 1.035 T. For a further confirmation, the measured magnetic fields at 1.084 T were scaled to 1.035 T and the calculated flux distribution was found to be a better fit to the measured data, shown in Fig. 18 ▸ (bottom row). The calculated field distribution of the fifth harmonic shows a very wide vertical distribution which could explain the asymmetric vertical distribution seen in the measured data.

## 5. Discussion

As it stands, Fig. 17 ▸ indicates that the flux of the fifth harmonic at 12 keV is 57% of the ideal which is a greater reduction than expected given the RMS phase error of 9.6° in Table 3 ▸. The impact of the reduction in the flux is such that the fifth and third harmonics at 12.4 keV are practically equivalent as seen in Fig. 18 ▸ (top row). The RMS phase error of 9.6° was calculated with a combination of field measurements and optimal AUX corrector strengths (numerically calculated). This degree of phase error should have resulted in a flux reduction of (72 ± 7)%, implying that improvements may still be possible by the further optimization of AUX corrector strengths. This is also somewhat supported by the fact that the spectrum calculated from the magnetic field data with the closest match to the measured flux distribution shows the fifth harmonic flux at 12.4 keV to be 27% higher than the third harmonic at 12.4 keV.

**Figure 19:**
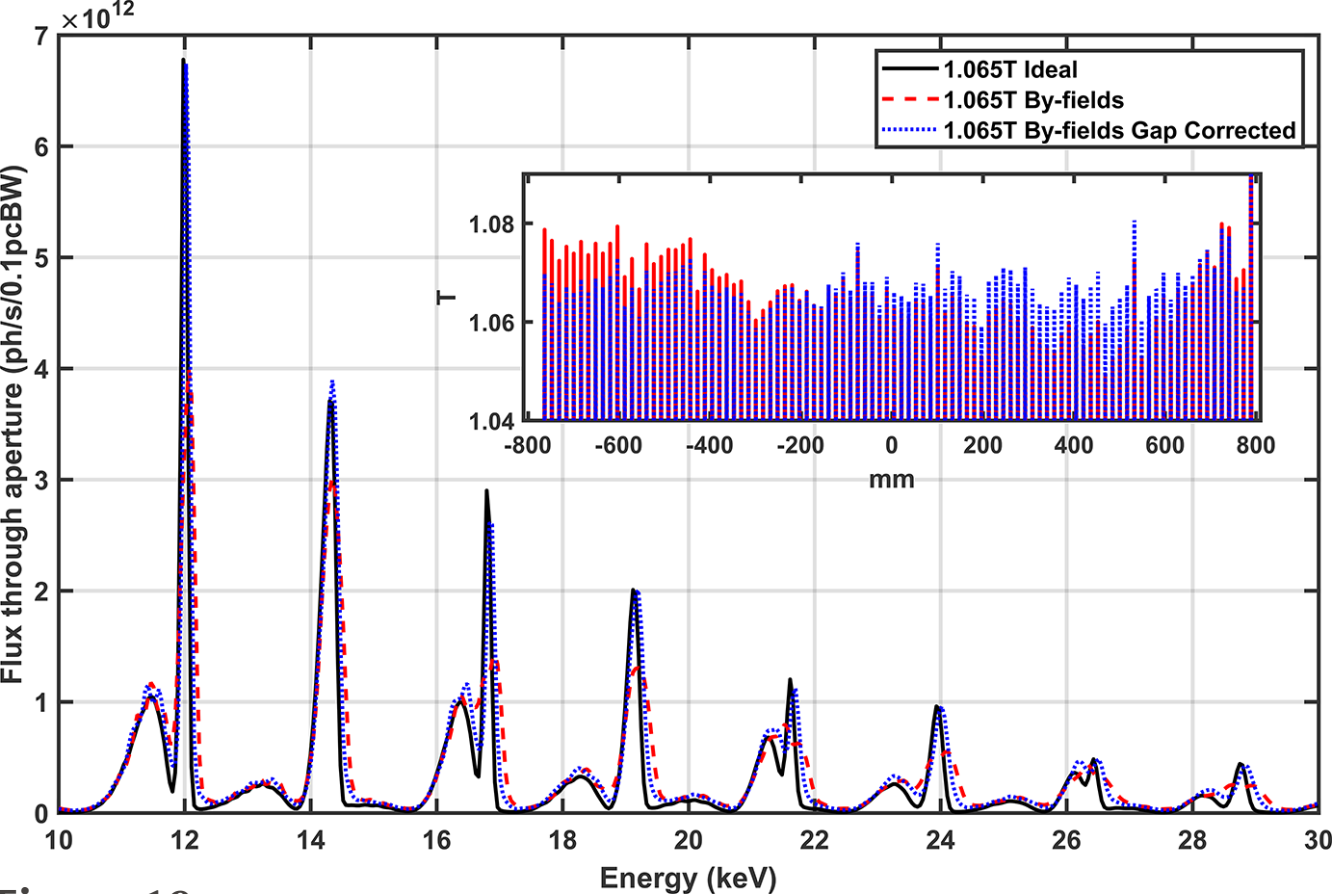
Comparison showing a near full recovery of the photon flux if the systematic gradient along the undulator could be corrected. The inset shows the measured magnetic field (red) and the same field linearly flattened field (blue) to mimic a small gap adjustment along the length of the magnet. The flattened field was use to calculate the ‘corrected’ spectrum.

The primary cause of the phase error is magnetic gap changes along the length of the array. The inset of Fig. 19 ▸ shows the field after the last iteration of mechanical shimming of the magnet where a quadratic component to the peak field along the length of the magnet array was observed. At the time it was unclear whether additional adjustments would improve or make it worse, and how long it would take. Each iteration took almost four weeks to complete. Time (and political) pressures forced the decision to stop. In hindsight this was unwise. Numerically, if the fields were scaled to correct the gap variation, the effective RMS phase error could be reduced to 5° and would have met the original requirement of <6°. Fig. 19 ▸ shows that much of the photon flux could be recovered if the systematic gradients were corrected.

This analysis shows that the field quality of the Bilfinger magnet array itself is very good and that the method of gap control was inadequate in this particular device.

At the time of writing, the beamline has been in operation for more than a year, taking on more than 200 users, and the SCU16 has been operating reliably. There are plans in place to repeat the AUX optimization and investigate the possibility of improving the flux by manipulating the vertical electron trajectory.

## Figures and Tables

**Table 1:** Ideal flux density [photons s^−1^ mrad^−2^ (0.1% bandwidth)^−1^] as a function of technology assuming the requirement of the fifth harmonic at 12.4 keV and a total length of 2.5 m, where *L*_m_ is the total magnet length

Type	*L*_m_ (m)	λ_u_ (mm)	*K*	Flux density	% change
HR SCU	1.6	15.6	1.583	5.44 × 10^16^	137
VR SCU	1.6	16.0	1.547	5.02 × 10^16^	126
CPMU	2.0	16.5	1.504	5.71 × 10^16^	143
CPMU	2.0	17.0	1.461	5.06 × 10^16^	127
RT IVU	2.0	18.0	1.381	3.98 × 10^16^	100

**Table 2:** Storage ring and SCU16 parameters

Parameter	Value
Electron energy	3.03 GeV
Natural emittance	10.5 nm rad
Coupling	1.24%
β_*x*,*y*_	8.92 m / 2.42 m
η_*x*,*y*_	0.10 m / 0.00 m
Total length	2.5 m
Magnet period	16.0 mm
Magnet length / periods	1.6 m / 98
Maximum field / *K*	1.084 T / 1.62
Maximum current	873 A
Magnet gap	8.0 mm
Vertical vacuum gap	5.6 mm
Horizontal vacuum gap	60.0 mm
Field stability (144 h)	<200 p.p.m.
Horizontal roll-off (±10 mm)	<0.35%
RMS peak field error	0.7%
RMS phase error	10°
Quench recovery time	25 min
Cool down / warm up	4.0 / 2.5 days

**Table 3:** Stretched wire measurements of the first (*I*1) and second (*I*2) field integrals and integrated quadrupole and skew quadrupole components (*k* and *k*_s_) Measurements were made with the upstream/downstream AUX2 and Helmholtz coils set to the values gained to minimize the integrals with the wire positioned at the beam axis (*X* = 0, *Y* = 0). Units: field in Tesla, I1 in 10^−6^ T m, I2 in 10^−6^ T m^2^ and *k* in mT.

Current (A)	Field (T)	*B*_*y*_*I*1	*B*_*y*_*I*2	*B*_*x*_*I*1	*B*_*x*_*I*2	*k*	*k*_s_	RMS phase error (°)
862	1.084	−0.32	−12.95	10.99	57.02	17	1.9	9.6
750	0.975	0.890	11.82	24.00	55.70	14	2.0	8.1
420	0.650	−7.48	30.17	−3.92	20.12	8	0.7	4.8
115	0.325	21.80	−64.89	−102.80	−146.59	2	0.2	1.7

**Table 4:** Lifetime before and after the installation of the SCU with the impact on the lifetime being less than 2% and no appreciable impact on the injection efficiency The initial impact on lifetime was largely due to increase in pressure due to outgassing of new vacuum chambers.

	Current lifetime	Vacuum	Lifetime change
Before	5.20 A h	4.5 × 10^−10^ mbar	
After	4.38 A h	1.9 × 10^−8^ mbar	84.5%
After	5.01 A h	2.0 × 10^−9^ mbar	98.8%

## Footnotes

1 Space in the straight was limited to a maximum of 2.5 m, and assumingsuperconducting devices requires ∼0.45 m on either side for transitions whileroom temperature undulators and CPMU would require ∼0.25 m.

2 Previously known as Bilfinger Noell GmbH.

3 The final design of the current monitoring will be a Danisense DM1200ID current sensor (1:1500 turns ratio) with a total accuracy of 6 p.p.m. and sampled by a Keithley DM6500 in current sensing mode.
